# Data on multimodal approach for early poor outcome (Cerebral Performance Categories 3-5) prediction after cardiac arrest

**DOI:** 10.1016/j.dib.2018.05.118

**Published:** 2018-05-25

**Authors:** Maenia Scarpino, Giovanni Lanzo, Francesco Lolli, Riccardo Carrai, Marco Moretti, Maddalena Spalletti, Morena Cozzolino, Adriano Peris, Aldo Amantini, Antonello Grippo

**Affiliations:** a SODc Neurofisiopatologia, Dipartimento Neuromuscolo-Scheletrico e degli Organi di Senso, AOU Careggi, Florence, Italy; b IRCCS, Fondazione Don Carlo Gnocchi, Florence, Italy; c Dipartimento di Scienze Biomediche Sperimentali e Cliniche, Università degli Studi di Firenze, Italy; d Neuroradiologia, AOU Careggi, Florence, Italy; e Unità di Terapia Intensiva, Dipartimento Neuromuscolo-Scheletrico e degli Organi di Senso, AOU Careggi, Florence, Italy

## Abstract

The data presented in this article are related to our research article entitled ‘Neurophysiological and neuroradiological multimodal approach for early poor outcome prediction after cardiac arrest’ (Scarpino et al., 2018) [1]. We reported two additional analyses, including results gathered from somatosensory evoked potentials(SEPs), brain computed tomography(CT) and electroencephalography(EEG) performed on 183 subjects within the first 24 h after cardiac arrest(CA). In the first analysis, we considered the Cerebral Performance Categories(CPC) 3, 4 and 5a,b (severe disability, unresponsive wakefulness state, neurological death and non-neurological death, respectively) as poor outcomes. In the second analysis, patients that died from non-neurological causes (CPC 5b) were excluded from the analysis. Concerning the first analysis, bilateral absent/absent-pathologic(AA/AP) cortical SEPs predicted poor outcome with a sensitivity of 49.3%. A Grey Matter/White Matter(GM/WM) ratio <1.21 predicted poor outcome with a sensitivity of 41.7%. Isoelectric/burst-suppression EEG patterns predicted poor outcome with a sensitivity of 33.5%. If at least one of these poor prognostic patterns was present, the sensitivity for an ominous outcome increased to 60.9%. Concerning the second analysis, AA/AP cortical SEPs predicted poor outcome with a sensitivity of 52.5%. GM/WM ratio <1.21 predicted poor outcome with a sensitivity of 50.4%. Isoelectric/burst-suppression EEG patterns predicted poor outcome with a sensitivity of 39.8%.

**Specifications Table**

**Table:** 

Subject area	*Cardiac Arrest(CA)*
More specific subject area	*Multimodal Neurological Prognosis*
Type of data	*Tables, text file, figures*
How data was acquired	*Retrospective observational*
Data format	*Analysed*
Experimental factors	*Electroencephalography(EEG) patterns classified according to the American Clinical Neurophysiology Society(ACNS) terminology; Somatosensory Evoked Potentials(SEPs) classified according to the cortical responses on both hemispheres; Grey Matter/White Matter(GM/WM) ratio density on Brain Computed Tomography(CT); neurological outcome evaluated using Cerebral Performance Categories(CPC)*
Experimental features	*All the three tests (EEG, SEPs and brain CT) were performed on surviving patients within the first 24 h after CA. The primary endpoint was neurological outcome at 6 months, assessed by CPC. Predictors were assessed using a Receiving Operating Characteristic(ROC) curve and classification tree.*
Data source location	*Florence, Italy*
Data accessibility	*The data are available with this article.*
Related research article	*Neurophysiological and Neuroradiological Multimodal Approach for early poor outcome prediction after Cardiac Arrest. Scarpino et al.,* Apr 18 (2018). doi: 10.1016/j.resuscitation.2018.04.016. [Epub ahead of print]**

**Value of the data**

● A description of which SEP, EEG and brain CT features are relevant for neurological prognostication in CA comatose surviving patients and a description of the statistical analysis.
● A statistical analysis, including patients with severe disability (CPC 3) in the poor outcome group (a neurological outcome aggregation similar to some of the previous studies).
● An additional statistical analysis, excluding patients who died from non-neurological causes (CPC 5b) from the poor outcome group (CPC 4–5).

## 1 Data

Prognosticating neurological outcome after CA is challenging and should require a multimodal approach that could be used to develop a prognostication algorithm.

## 2 Experimental design, materials, and methods

### 2.1 Patient management

All CA comatose patients included in the analysis underwent to brain CT upon admission to the emergency room, and EEG and SEP evaluation within the first 24 after CA. The results of these instrumental tests did not affect ongoing patient management, in fact in our clinical practice the withdrawal of life support was not included and treatments were not suspended except where patients with confirmed brain death (BD) were concerned. Patient discharge to a long-term care unit or to a rehabilitation unit was decided, in agreement with the intensivists, according to these neurophysiological and neuroradiological findings associated with the clinical data. Neurological status was determined using CPC at two follow-up points: at hospital discharge, looking at the chart review, and, for patients surviving at hospital discharge, at least 6 months after CA, by telephone interview.

#### 2.1.1 SEP, EEG and brain CT evaluation

SEP prognostic power was based on the evaluation of the presence/absence or of the amplitude value of the cortical responses (N20/P25 complex) on both hemispheres. Thus, we identified these six SEP patterns: NN, NP, PP, AN, AP and AA, in which N stands for normal (N20/P25 amplitude is normal), P stands for pathological (N20/P25 amplitude is <1.2 µV or the difference between the two sides is greater than 50%) and A stands for absent, if no reproducible cortical components could be identified in the presence of a cervical potential [1], [2], [3].

EEGs were classified according to the terminology for EEGs recorded in ICU [4]. The continuity and the voltage of the background activity were the main parameters taken in to account for EEG classification. Thus, the main patterns identified were: continuous; nearly continuous; discontinuous; burst-suppression; suppression; epileptiform discharges, low voltage (voltage <20 μV) and isoelectric. Isoelectric (voltage <2 μV) recordings were identified, although the original classification did not distinguish them from suppressed activity (voltage <10 μV) [5].

Brain CT prognostic power is based on the GM/WM ratio as a measure of density. In particular, in our analysis, we performed density measurements limited to the basal ganglia level, according to a previously reported method [6], as the GM/WM ratio=(caudate nucleus+putamen)/(corpus callosum+posterior limb of the internal capsule).

For further details regarding SEP and EEG recording and brain CT acquisition, refer to the supplementary data of the related research article [1].

#### 2.1.2 Statistical analysis

We used the receiver operating characteristic (ROC) to determine the sensitivity at a specificity of 100% (false positive rate=0%) for SEPs, EEG, and GM/WM ratio, in relation to poor outcome. We expressed the performance of each measure for predicting poor outcome as the area under the ROC curve (AUC). The dependent variable, outcome, was dichotomous (good/poor). Two set of analysis were performed. In the first one, we considered as a good outcome no or minor neurological deficits (CPC=1) and moderate disability (CPC=2), and as a poor outcome severe disability (CPC=3), unresponsive wakefulness state (CPC=4) and death (CPC=5a-b). In the second analysis, having distinguished patients who died from neurological causes (CPC=5a) from those who died from non-neurological causes (CPC=5b), according to the suggestion of Sandroni et al. [7], we evaluated the predictive value of the three tests in all patients except in those that died from non-neurological causes. Then, in poor outcome, group we included subjects with CPC=4 and CPC=5a. A *p*-value <0.05 was considered statistically significant. Statistical analysis was performed using the Stat-View Software package (SAS Institute).

## 3 Results

Table 1 shows the demographic characteristics of the 183 subjects that were subjected to all three tests within the first 24 h after CA.

**Table 1:** Patient baseline demographics and outcome.

	Patients included *n*=183
Mean age (yrs) mean (SD)	66.0 (15.9)
Male (%)	120 (65.5)
Out-of-hospital arrest (%)	128 (69.9)
Witnessed arrest (%)	155 (84.6)
CA duration (min) median (IQR)	24.2 (14)
**Initial rhythm**	
VF/VT (%)	78 (42.7)
PEA/EMD (%)	47 (25.6)
Asystole (%)	38 (20.7)
Unknown (%)	20 (10.9)
**Pupillary reflex at NPH evaluation (%)**	
Yes	36 (19.6)
No	140 (76.5)
NA	7 (3.8)
**GCS score at ICU admission**	
Total median (IQR)	3.0 (0.0)
Motor median (IQR)	1.0 (0.0)
Verbal median (IQR)	1.0 (0.0)
Eyes median (IQR)	1.0 (0.0)
**CPC score**	
***Discharge***	
CPC 1, good recovery (%)	5 (2.7)
CPC 2, moderate disability (%)	12 (6.5)
CPC 3, severe disability (%)	33 (18.0)
CPC 4, unresponsive wakefulness state (%)	72 (39.3)
CPC 5a, brain death (%)	34 (18.5)
CPC 5b, death from non neurological causes (%)	27 (14.7)
***6 months***	
CPC 1, good recovery (%)	9 (4.9)
CPC 2, moderate disability (%)	28 (15.3)
CPC 3, severe disability (%)	23 (12.5)
CPC 4, unresponsive wakefulness state (%)	54 (29.5)
CPC 5a, brain death (%)	34 (18.5)
CPC 5b, death from non neurological causes (%)	35 (19.1)

Yrs, years; SD, standard deviation; n, number; ICU, intensive care unit;

CA, cardiac arrest; min, minutes; IQR, interquartile range; VF, ventricular

fibrillation; VT, ventricular tachycardia; PEA, pulseless electrical activity;

EMD, electromechanical dissociation; NA, not available; NPH, neurophysiological;

GCS, Glasgow Coma Scale; TTM, Targeted Temperature Management;

CPC, Cerebral Performance Categories

### 3.1 Single parameter approach at 100% specificity for poor outcome prediction (CPC 3-4-5a-5b)

**Fig. 1:**
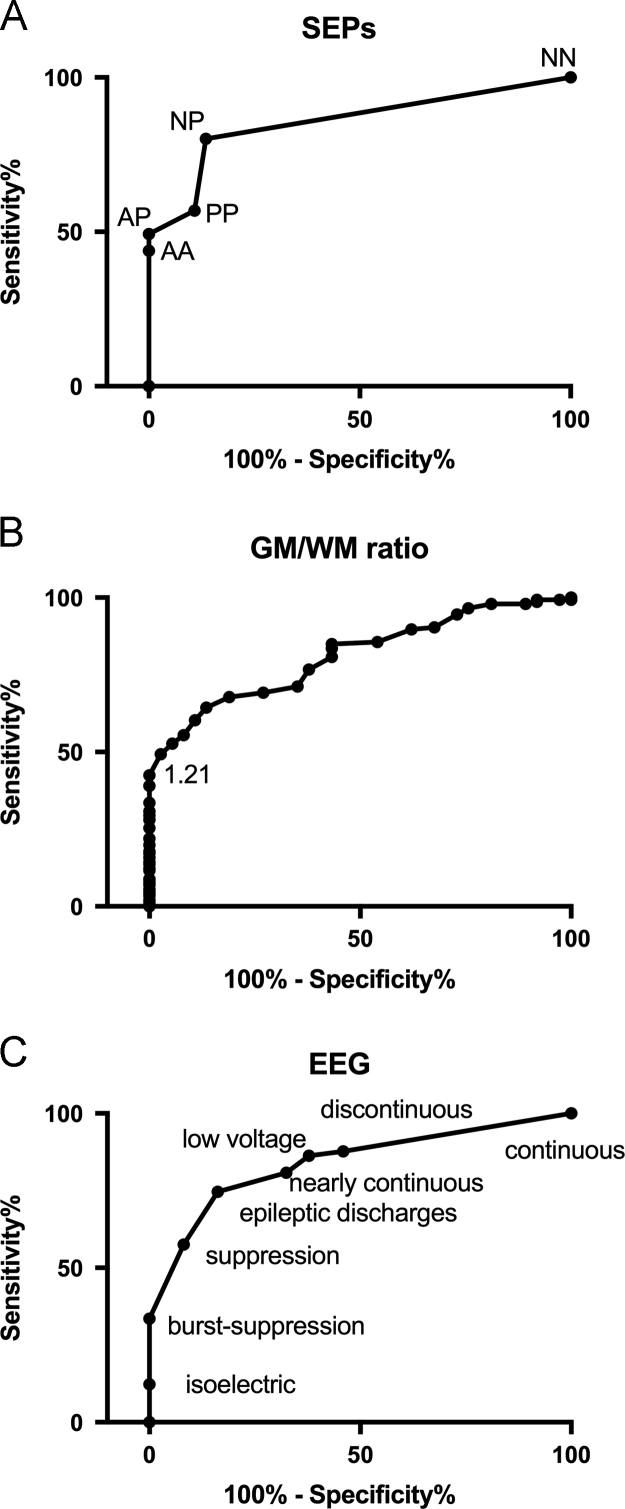
ROC curves showing accuracy in the prediction of a poor prognosis in comatose patients after CA, in which CPC 3, CPC 4 and CPC 5a,b were all included in the poor outcome group, according to SEP findings (A), the GM/WM ratio (B) and EEG patterns (C). The ordinate axis shows the sensitivity of the tests, ranging from 0 to 1.0 (0–100%), while the abscissa shows the percentage of false positive results (100% specificity). Tests with good discriminatory power produced an ROC curve that closely follows the left-hand axis and the top margin of the graph, passing close to the upper left corner.

According to the ROC analysis (AUC=0.85/Confidence Interval (CI) 0.79–0.91, Fig. 1A), the SEP patterns could be combined in a two-graded scale: grade 1 (NN-NP-PP) and grade 2 (AA-AP). Grade 2 SEPs predicted a poor outcome with a sensitivity of 49.3% for a specificity of 100% (CI 90.5–100). Concerning the GM/WM ratio, ROC analysis demonstrated an AUC=0.81 (CI 0.74–0.87), as shown in Fig. 1B. A GM/WM ratio <1.21 predicted a poor outcome with a sensitivity of 41.7% for a specificity of 100% (CI 90.5–100). According to the ROC analysis (AUC=0.83/CI 0.77–0.90, Fig. 1C), EEG patterns could be combined in a two-graded scale: non-malignant (suppression, discontinuous, nearly-continuous, continuous, epileptiform discharges, low voltage) and malignant (isoelectric, burst-suppression) patterns. Malignant EEG patterns predicted a poor outcome with a sensitivity of 33.5% for a specificity of 100% (CI 90.5–100). Table 2 shows the sensitivity and NPV of EEG, SEPs and GM/WM ratio for the whole study cohort.

**Table 2:** Single and multimodal approach-sensitivity and negative predictive values (at 100% specificity) for poor outcome prediction.

**Parameter**	**CPC 3–4-5a-5b “poor”**	**CPC 1–2 “good”**	**Sensitivity**	**NPV**
**Single Test**			95% CI	95% CI
**SEPs**				
**Grade 2**	72	0	49.3% (40.9–57.7)	33.3% (29,8–36.9)
**Grade 1**	74	37		
**GM/WM ratio**				
**<1.21**	61	0	41.7% (33.6–50.2)	35.6.5% (32.5.1-38.8)
**≥1.21**	85	37		
**EEG**				
**Malignant**	49	0	33.5% (25.9–41.8)	27.6% (25.3–29.9)
**Non Malignant**	97	37		
**Multimodal**				
**Different Combination of two tests**				
**Grade 2 SEPs or GM/WM ratio < 1.21**	84	0	57.7% (49.0–65.6)	43.1% (38.5–47.8)
**Grade 1 SEPs or GM/WM ratio ≥1.21**	62	37		
**Malignant EEG or GM/WM ratio < 1.21**	81	0	55.4% (47.0-63.7)	41.9% (37.6–46.4)
**Non Malignant EEG or GM/WM ratio ≥1.21**	65	37		
**Grade 2 SEPs or Malignant EEG**	71	0	48.6% (40.2–57.3)	33.0% (29.6–36.6)
**Grade 1 SEPs or Non Malignant EEG**	75	37		
**Combination of three tests**				
**One or more tests predicting poor outcome**	89	0	60.9% (52.5–68.9)	39.3% (34.6–44.2)
**No test predicting poor outcome**	57	37		

CPC: Cerebral Performance Categories; NPV: Negative Predictive Value; CI: Confidence Interval; SEPs: Somatosensory Evoked Potentials; GM/WM: Gray Matter/White Matter.

### 3.2 Multimodal approach at 100% specificity for poor outcome prediction (CPC 3-4-5a-5b)

After determining the optimal cut-off for a specificity of 100% for each parameter, we combined the results of the three tests in the hypothesis that the ominous prognostic findings of each test were not all present simultaneously in the same patient, thus evaluating whether the availability of more than one test in the same patient could increase the predictability of a poor outcome. Actually, 89 patients had at least one poor prognostic parameter (grade 2 SEPs, malignant EEG patterns, GM/WM ratio <1.21). When two tests were considered, if at least one of the patterns predicting a poor outcome was present, the sensitivity increased by 49.3% (obtained with the best single performing test, SEPs) reaching a maximum of 57.7% (obtained by SEPs and brain CT combination). Finally, when all three tests were considered, if at least one of the patterns predicting a poor outcome was present, the sensitivity for a poor prognosis increased to a maximum of 60.9%.

### 3.3 Single parameter approach at 100% specificity for poor outcome prediction (CPC 4–5a) with the exclusion of patients who died from non-neurological causes (CPC 5b)

**Fig. 2:**
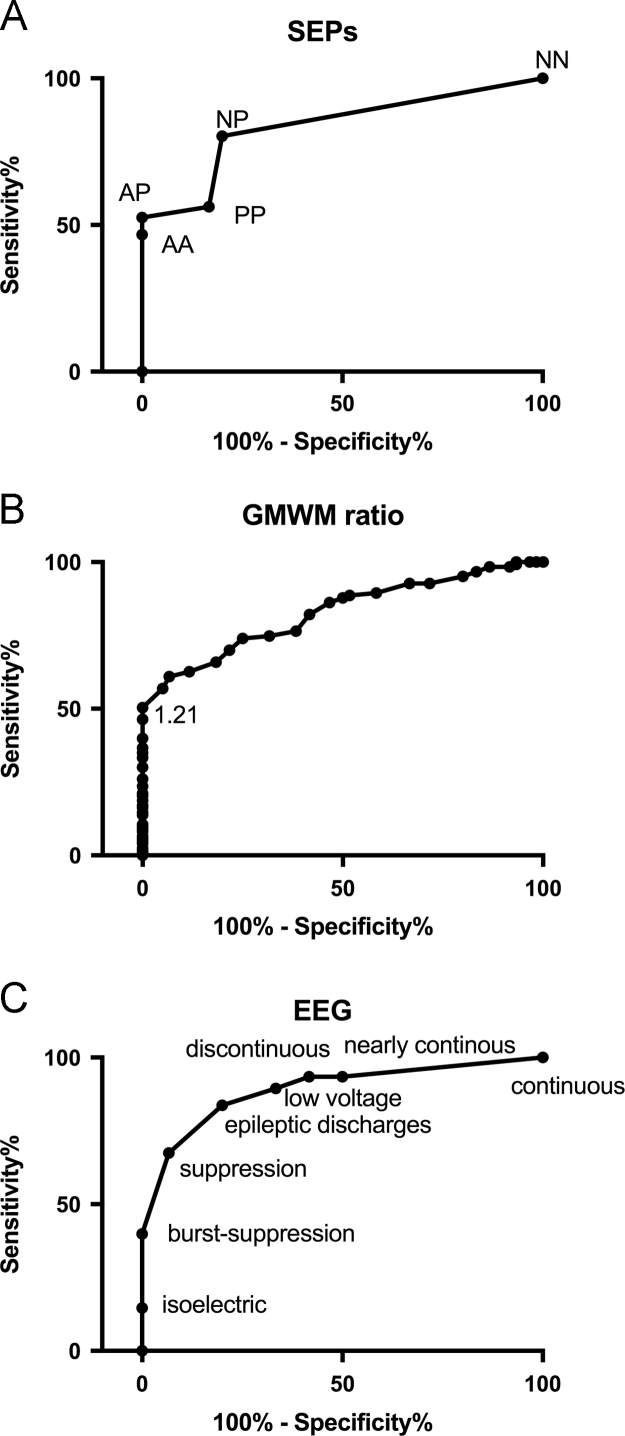
ROC curves showing accuracy in the prediction of a poor prognosis in comatose patients after CA, excluding patients who died from non-neurological causes, thus considering CPC 4 and CPC 5a in the poor outcome group, according to SEP findings (A), the GM/WM ratio (B) and EEG patterns (C). The ordinate axis shows the sensitivity of the tests, ranging from 0 to 1.0 (0–100%), while the abscissa shows the percentage of false positive results (100% specificity). Tests with good discriminatory power produced an ROC curve that closely follows the left-hand axis and the top margin of the graph, passing close to the upper left corner.

According to the ROC analysis (AUC=0.83/CI 0.77–0.89, Fig. 2A), the SEP patterns could be combined in a two-graded scale: grade 1 (NN-NP-PP) and grade 2 (AA-AP). Grade 2 SEPs predicted a poor outcome with a sensitivity of 52.5% (CI 43.8–61.1) for a specificity of 100% (CI 94.0–100). Concerning the GM/WM ratio, ROC analysis demonstrated an AUC=0.82 (CI 0.76–0.88), as shown in Fig. 2B. A GM/WM ratio <1.21 predicted a poor outcome with a sensitivity of 50.4% (CI 41.2–59.5) for a specificity of 100% (CI 94.0–100). According to the ROC analysis (AUC=0.88/CI 0.84–0.93, Fig. 2C), EEG patterns could be combined in a two-graded scale: non-malignant (suppression, discontinuous, nearly-continuous, continuous, epileptiform discharges, low voltage) and malignant (isoelectric, burst-suppression) patterns. Malignant EEG patterns predicted a poor outcome with a sensitivity of 39.8% (CI 31.1–49.0) for a specificity of 100% (CI 94.0–100).
